# Active and Passive Immunization with rHyr1p-N Protects Mice against Hematogenously Disseminated Candidiasis

**DOI:** 10.1371/journal.pone.0025909

**Published:** 2011-10-10

**Authors:** Guanpingsheng Luo, Ashraf S. Ibrahim, Samuel W. French, John E. Edwards, Yue Fu

**Affiliations:** 1 Division of Infectious Diseases, Los Angeles Biomedical Research Institute at Harbor-UCLA Medical Center, Torrance, California, United States of America; 2 David Geffen School of Medicine at UCLA, Los Angeles, California, United States of America; 3 Department of Pathology, Los Angeles Biomedical Research Institute at Harbor-UCLA Medical Center, Torrance, California, United States of America; Albert Einstein College of Medicine, United States of America

## Abstract

We previously reported that *Candida albicans* cell surface protein Hyr1 encodes a phagocyte killing resistance factor and active vaccination with a recombinant N-terminus of Hyr1 protein (rHyr1p-N), significantly protects immunocompetent mice from disseminated candidiasis. Here we report the marked efficacy of rHyr1p-N vaccine on improving the survival and reducing the fungal burden of disseminated candidiasis in both immunocompetent and immunocompromised mice using the FDA-approved adjuvant, alum. Importantly, we also show that pooled rabbit anti-Hyr1p polyclonal antibodies raised against 8 different peptide regions of rHyr1p-N protected mice in a hematogenously disseminated candidiasis model, raising the possibility of developing a successful passive immunotherapy strategy to treat this disease. Our data suggest that the rabbit anti-Hyr1p antibodies directly neutralized the Hyr1p virulence function, rather than enhanced opsonophagocytosis for subsequent killing by neutrophil *in vitro*. Finally, the rHyr1p-N vaccine was protective against non-*albicans Candida spp*. These preclinical data demonstrate that rHyr1p-N is likely to be a novel target for developing both active and passive immunization strategies against *Candida* infections.

## Introduction

*Candida* species, the third most common cause of healthcare-associated bloodstream infections [1] causes approximately 60,000 cases of hematogenously disseminated candidiasis per year in the United States [2], resulting in billions of dollars of healthcare expenditures. Notwithstanding current antifungal therapy [3], [4], [5], mortality remains unacceptably high [6], [7], [8]. Because of the rising incidence of life-threatening candidiasis and high treatment failure rates, more effective prophylactic and therapeutic strategies are needed.

*HYR1* belongs to the *IFF* gene family of *C. albicans*, which includes 12 members [9]. It encodes a cell surface glycosylphosphatidylinositol (GPI)-anchored protein that is expressed during hyphal formation [10], [11]. In our previous study, we showed that Hyr1p mediated *C. albicans* resistance to phagocyte killing *in vitro* and contributed higher fungal burden in organs rich in phagocytes (e.g. liver and spleen) [12]. Native *HYR1* is positively regulated by transcription factor Bcr1p [13]. We found that autonomous *HYR1* expression reversed the hyper-susceptibility to phagocyte-mediated killing of a *bcr1* null mutant of *C. albicans in vitro* [12]. Further, heterologous expression of *HYR1* in *C. glabrata* rendered the organism more resistant to phagocyte killing [12]. Our study also showed that a vaccine based on the recombinant N terminus of Hyr1p (rHyr1p-N) markedly improved survival of immunocompetent mice challenged intravenously with *C. albicans* when mixed with either Freund's or alum as an adjuvant [12].

The current studies were performed to further define the vaccine efficacy of rHyr1p-N vaccine in both immunocompetent and immunocompromised mice using the FDA-approved alum as an adjuvant. Further, the breadth of protection induced by rHyr1p-N was evaluated by its efficacy against non-*albicans Candida* species. Finally, we sought to study the potential use of passive immune therapy in disseminated candidiasis using anti-Hyr1p antibodies.

## Results

### The rHyr1p-N vaccine significantly improved survival and decreased fungal burden in immunocompetent mice challenged intravenously with C. albicans

**Figure 1:**
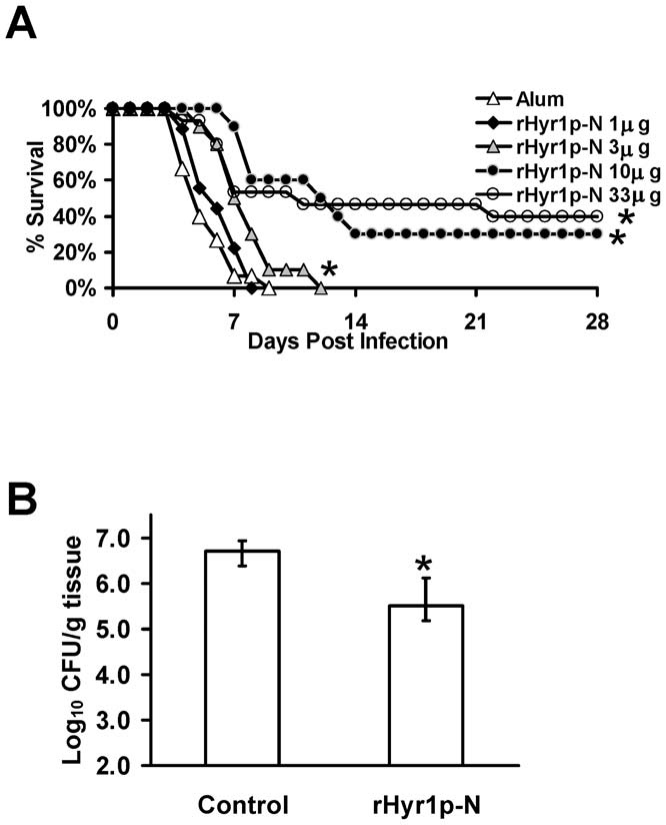
rHyr1p-N vaccine improved survival and decreased fungal burden in mice with *C. albicans* infection. (A) Survival of vaccinated or control mice (n = 15 per group) infected intravenously with *C. albicans* 15563 strain, a clinical isolate (9×10^5^ per dose), * *P*<0.001 compared to alum alone by the log-rank test. (B) Kidney fungal burden of mice (n = 10 per arm) vaccinated with 33 µg rHyr1p-N + alum or alum alone and harvested 3 days post infection with *C. albicans* 15663 (7×10^5^ per dose). Data are presented as median ± interquartile ranges. * *P*<0.001 compared to results obtained from kidneys harvested from mice vaccinated with alum alone by the Mann-Whitney U test.

To determine the most effective dose of the rHyr1p-N immunogen, an approximately 3-fold dose range was evaluated (1 to 33 µg per mouse). Female juvenile BALB/c mice were immunized with rHyr1p-N plus alum (2% Alhydrogel; Brenntag Biosector) or with alum alone. These mice were subsequently infected with a lethal inoculum of *C. albicans* (7×10^5^ blastospores). Vaccinated mice had significant improvements in survival compared to adjuvant control mice (Figure 1A). All tested doses, except 1 µg, prolonged or improved survival compared to mice receiving adjuvant alone, and a dose response was found with 10 and 33 µg having the greatest efficacy (Figure 1A). The experiment was terminated on day 28, with all remaining mice appearing healthy.

To determine the impact of vaccination on fungal burden, juvenile mice were vaccinated and infected as above. On day 3 post-infection (one day before the control mice were predicted to die based on the previous experiment), mice were euthanized and kidneys, being the primary target organ, were harvested to determine tissue fungal burden. Vaccination reduced the tissue fungal burden by approximately 16-fold compared to control mice (p<0.001) (Figure 1B).

**Figure 2:**
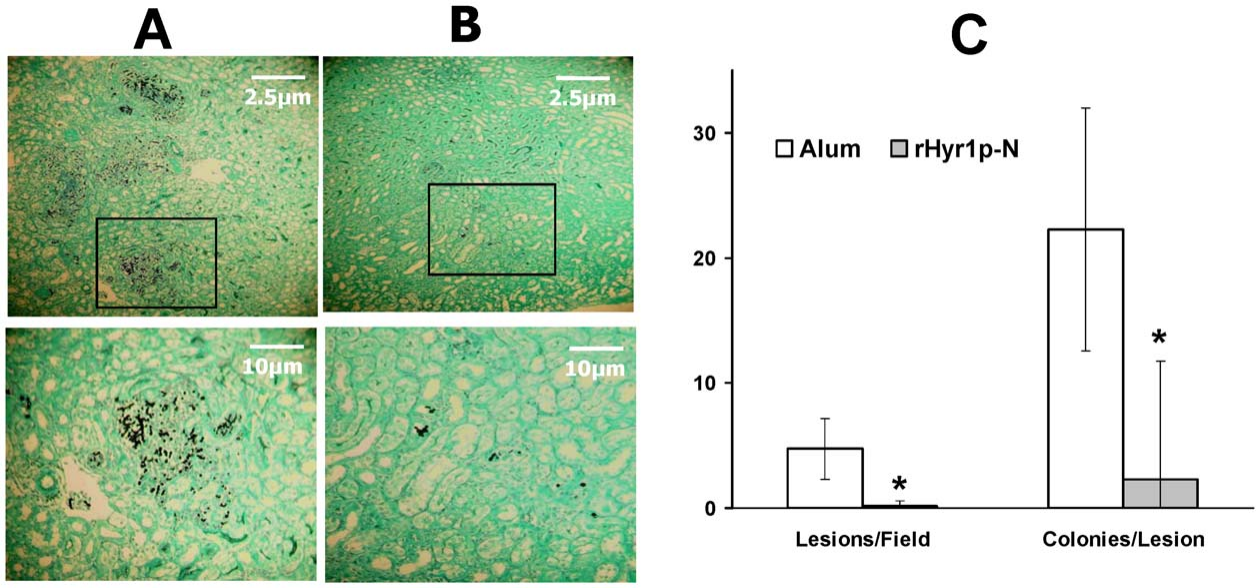
Representative histopathological sections from kidneys were shown. (A) Control mice infected with *C. albicans* had multiple abscesses showing mostly yeast forms with some hyphae and pseudohyphae throughout the kidneys. (B) rHyr1p-N vaccinated mice (33 µg) infected with *C. albicans* had less abscesses with far less fungi visible. (C) Semiquantitative evaluation of the severity of infection indicated significant abscess and *Candida* cells reduction in vaccinated mice compared to control mice. Sections were stained by PAS. Thirty random fields were examined by a blinded assessor (GL) to assess the number of lesions per field. Number of organisms per lesion was evaluated in 120 lesions in the control unvaccinated mice. The average number of organisms per lesion was determined by dividing the total number of fungal cells by the number of lesions counted. * *P*<0.0001 by Wilcoxon rank sum test.

Consistent with the survival and fungal burden data, histopathological examination of kidneys harvested from rHyr1p-N vaccinated mice demonstrated very few abscesses with minimal fungal residues mainly present in the blastospore formation (Figure 2B). However, numerous abscesses full of fungal cells showing mostly yeast forms with some hyphae and pseudohyphae were detected in kidneys taken from mice vaccinated with alum alone (Figure 2A). Semi-quantitative evaluation of the severity of infection showed a significant reduction of abscesses per field as well as reduced *Candida* cells per abscess in vaccinated mice compared to that in controls (Fig. 2C, *P*<0.0001).

### The rHyr1p-N effectively protected immunocompromised mice against candidiasis

**Figure 3:**
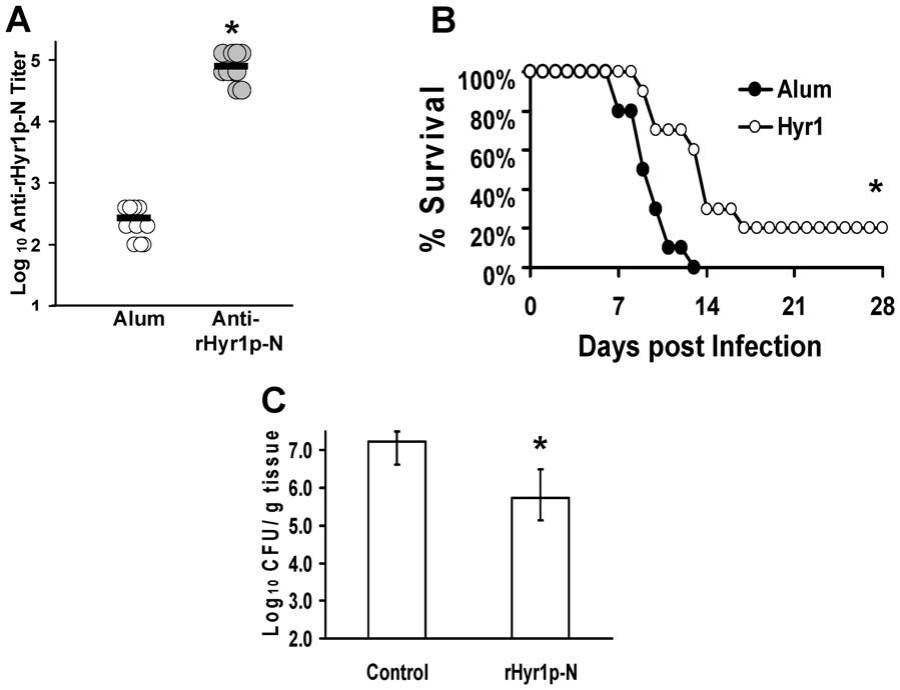
rHyr1p-N vaccine prolonged survival and decreased fungal burden in neutropenic mice infected with *C. albicans*. Balb/c mice (n = 20 per arm) were vaccinated with rHyr1p-N mixed with alum or alum alone (control), treated with cyclophosphamide, and then infected with *C. albicans* 15563 at 1×10^5^ blastospores. Two days before cyclophosphamide treatment, half of the mice were bled and individually marked for antibody titer using ELISA (A) (rHyr1p-N vaccinated versus control, *P = 1.08E-05 by Wilcoxon rank-sum test) and survival (B) (rHyr1p-N vaccinated versus control, *P = 0.007 by log rank test). The other half mice were used for fungal burden (C) * *P* = 0.002 by Wilcoxon rank-sum test.

It is known that a significant fraction of immunocompromised patients do respond to a variety of vaccines [14], [15], [16], [17]. We sought to define the potential usage of the rHyr1p-N vaccine to protect neutropenic mice from disseminated candidiasis. Immunized mice were bled twelve days following the boost with 30 µg of rHyr1p-N. Vaccination significantly increased the mouse immune response as determined by detection of increased anti-rHyr1p-N antibody titers (*P* = 1.08E-05) (Figure 3A). One day after the bleeding, mice were made neutropenic. Vaccination resulted in significant improvements in survival (*P* = 0.007 versus control) (Figure 3B).

We also evaluated the kidney fungal burden on day 10 post infection. Concordant with our survival result, we found that mice vaccinated with 30 µg of rHyr1p-N had 1.50 log fold decrease in fungal burden compared to kidneys harvested from control mice (Figure 3C, *P* = 0.002).

### Passive immunization with anti-Hyr1p IgG prolonged the survival of mice infected with C. albicans

**Figure 4:**
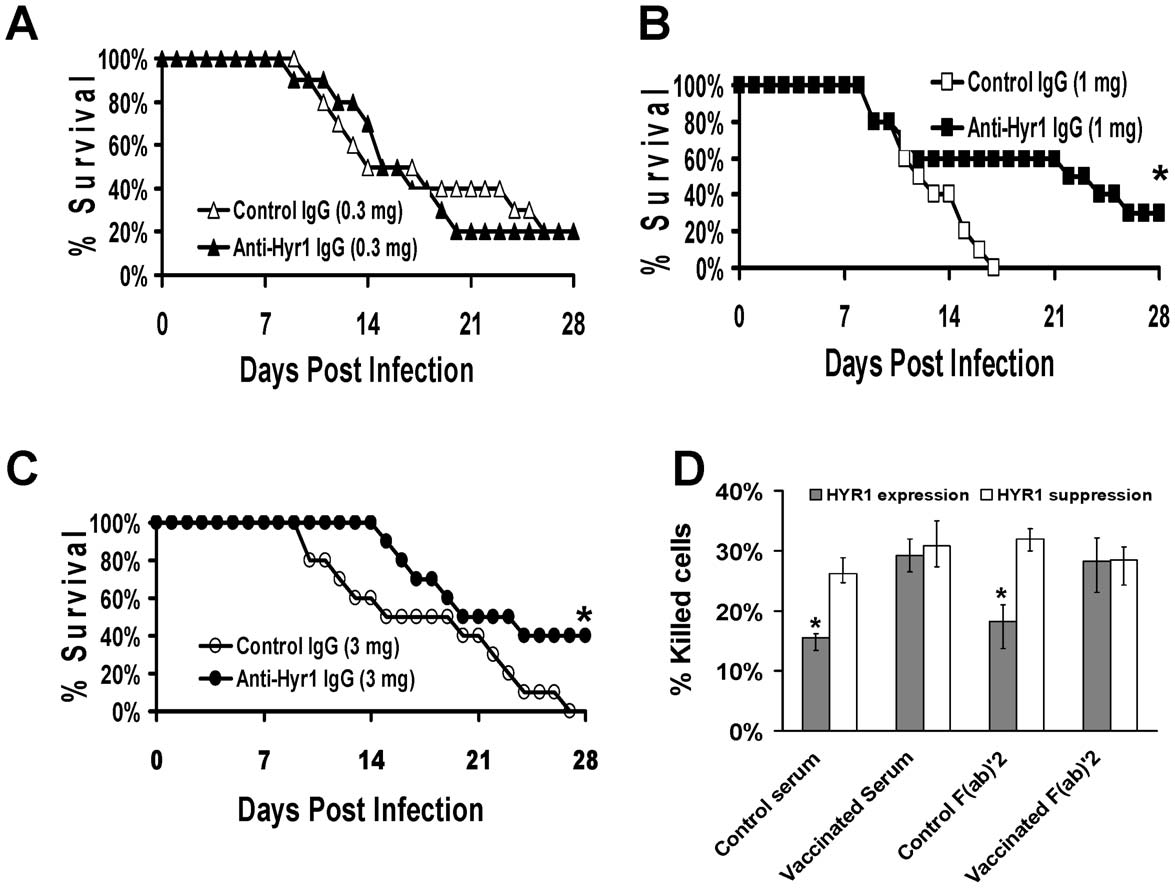
Dose dependent passive immunization with anti-Hyr1p IgG protected against murine hematogenously disseminated candidiasis. Mice were given 0.3 mg (A), 1 mg (B) and 3 mg (C) of anti-Hyr1p IgG by intraperitoneal injection 2 hr before infecting with 6.2×10^5^ blastospores of *Candida albicans* 15563 via the tail vein. Survival of mice (n = 10 per group) was monitored twice daily. * *P* = .001 by log-rank test vs. mice receiving non-specific, rabbit control IgG. (D) Effect of vaccinated or control F(ab′) _2_ on blocking HL-60 derived neutrophil killing of *C. albicans. C. albicans* overexpressing or suppressing Hyr1p were used in the assay to demonstrate specificity of the F(ab′) _2_ fragments to Hyr1p. Control denotes assay performed either in the absence of F(ab′) _2_ or in the presence of F(ab′)_2_ from non-specific, rabbit control IgG. Data are displayed as median ± interquartile range. * *P* = .001 by Mann-Whitney test.

Since some patients might not respond to an active vaccine strategy, we evaluated the possibility of using passive immunotherapy targeting Hyr1p. We generated polyclonal antibodies by vaccinating rabbits with 8 hydrophilic, highly antigenic 14-mer peptides located within rHyr1p-N region (Table 1). Purified IgG targeting these 8 peptides were pooled and used to treat naïve mice infected with a lethal dose of *C. albicans*. Mice receiving anti-Hyr1p IgG at either 1 or 3 mg (but not when administered at 0.3 mg) were protected substantially from infection when compared to mice receiving non-specific, rabbit control IgG from commercial source (Figure 4A, 4B and 4C).

**Table 1:** Hyr1 peptides used in this study.

Peptide Number	Sequence	MW (kDa)	pI	Purity (%)	Source
1	CGPSAPESESDLNTP	1.5	3.44	86.1	This study
2	CGNRDHFRFEYYPDT	1.9	5.69	99.4	This study
3	CGYDSKLFRIVNSRG	1.7	9.16	95.7	This study
4	CKIKGTGCVTADEDT	1.5	4.70	86.4	This study
5	CLKNAVTYDGPVPNN	1.6	6.25	94.1	This study
6	NSKSSTSFSNFDIGC	1.6	6.25	91.4	This study
7	CEPTHNFYLKDSKSS	1.8	7.19	85.8	This study
8	TSRIDRGGIQGFHGC	1.6	8.27	91.8	This study

Additional cysteine residues on the N- or C-termini were used to conjugate the 14-mer peptide to KLH.

To determine if the generated anti-Hyr1p antibodies enhanced phagocyte function by increasing opsonophagocytosis or by neutralizing Hyr1 killing resistance, we isolated and prepared F(ab′) _2_ fragments from pooled IgG raised against the 8 peptides of Hyr1p (conjugated to keyhole limpet hemocyanin or KLH) or from non-specific, rabbit control IgG. These fragments were used in HL-60 derived neutrophil killing assay against *C. albicans* conditionally overexpressing or suppressing Hyr1p rather than wild-type *C. albicans* to demonstrate specificity of these fragments to Hyr1p and not to other members of IFF family [9]. Consistent with our previous mouse IgG data [12], we found that F(ab′) _2_ fragments prepared from anti-Hyr1p antibodies but not those prepared from control antibodies were able to restore HL-60 derived neutrophil killing of the *HYR1* conditional expressing strain to levels equivalent to that of the suppressing strain (Figure 4D).

**Figure 5:**
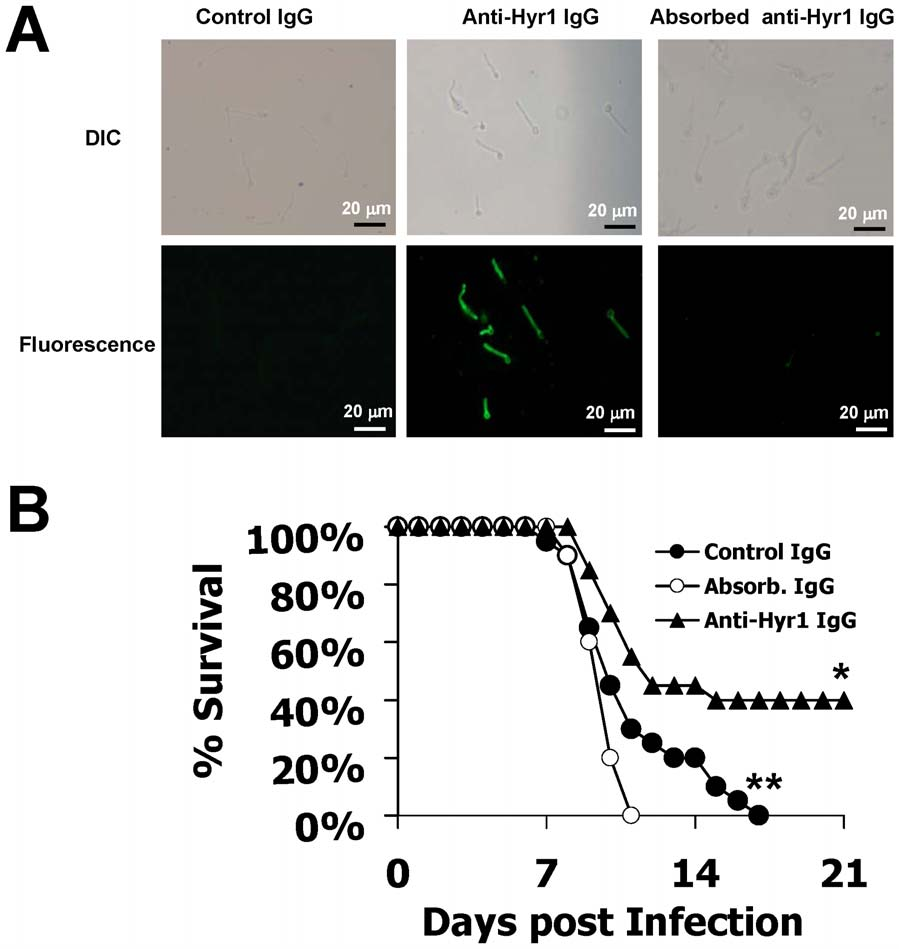
Protection against hematogenously disseminated candidiasis using purified pooled IgG was specific to Hyr1p. A) Indirect immunofluorescence with rabbit anti-Hyr1p IgG demonstrated surface expression of Hyr1p on *C. albicans* hyphae and the successful absorption of anti-Hyr1p antibodies; B) Survival of mice treated with 1 mg of: 1) pooled anti-Hyr1p IgG (n = 20); 2) pooled anti-Hyr1p IgG absorbed with *C. albicans* hyphae (n = 10); or 3) rabbit control IgG (n = 20) 2 hr before infecting with 8.7×10^5^ blastospores of *C. albicans* 15563 via the tail vein. The antibody dose was repeated 3 days after infection. * *P* = 0.002 for pooled anti-Hyr1p IgG vs. absorbed IgG, * *P* = 0.03 for pooled anti-Hyr1p IgG vs. control IgG, ** *P* = 0.28 for control IgG vs. absorbed IgG by Log Rank test.

To verify that the protection elicited by antibodies was indeed due to anti-Hyr1p antibodies and not due to non-specific protection caused by antibodies reacting to unrelated immunogen such as peptide carrier protein KLH, the purified IgG targeting the 8 hydrophilic rHyr1p-N peptides was absorbed with *C. albicans* hyphae prior to testing for their protective activity against hematogenously disseminated candidiasis. The absorbed IgG did not stain *C. albicans* hyphae (Figure 5A), indicating the anti-Hyr1p IgG were successfully eliminated. Furthermore, similar to non-specific, rabbit control IgG, the absorbed IgG did not protect mice from *C. albicans* infection, whereas the purified, non-absorbed IgG did (Figure 5B, *P* = 0.002).

### The rHyr1p-N vaccine substantially reduced tissue fungal burden in BALB/c mice challenged with several non- albicans species of Candida

**Figure 6:**
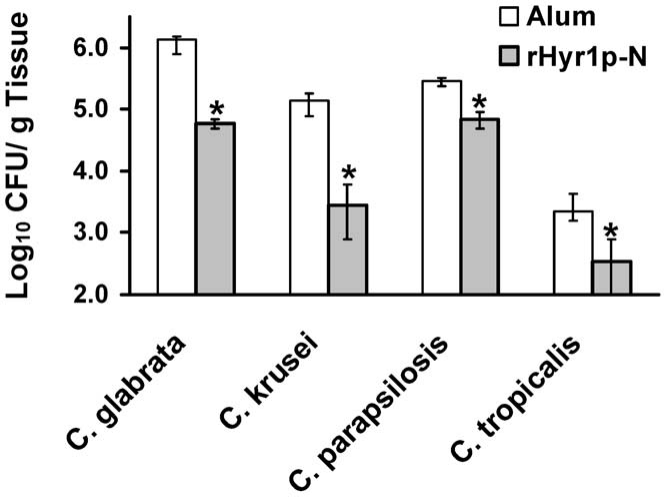
rHyr1p-N vaccine reduces tissue fungal burden in BALB/c mice infected with non-*albicans* species of *Candida*. BALB/c mice (n = 10 per group) were vaccinated with alum or alum plus rHyr1p-N (30 µg) and boosted three weeks later. Two weeks after the boost, mice were challenged via the tail vein with *C. glabrata* (3.2×10^7^), *C. krusei* (3.4×10^7^), *C. parapsilosis* (9.6×10^6^), or *C. tropicalis* (3.2×10^6^). Kidney fungal burden was determined on day 3 post infection. The *y* axis reflects the lower limit of detection of the assay. * *P*<0.001 versus adjuvant control by the Mann-Whitney U test.

A vaccine that elicits protection against *C. albicans* and other non-*albicans* species is highly desirable because a significant number of *Candida* infections are caused by non-*albicans* species. For example, *C. glabrata* represents the second most common cause of candidiasis and *C. krusei* is resistant to azole therapy. Using blast searches we were able to detect Hyr1p like molecules in several *Candida* species with amino acid similarity ranging between 47–72% in certain areas. Thus, we vaccinated mice with rHyr1p-N plus alum as above, then challenged with *C. albicans*, *C. glabrata*, *C. krusei*, *C. parapsilosis*, or *C. tropicalis*. Three days post infection mice were sacrificed and the kidneys harvested for determination of tissue fungal burden through colony counts. Mice vaccinated with rHyr1p-N had 0.65–1.69 log decrease in kidney fungal burden compared to mice vaccinated with alum alone (Figure 6, *P*<0.001).

## Discussion

*C. albicans* vaccine development has focused on using cell surface components [18], [19], peptides derived from cell wall proteins as immunogens [20], or on antibodies targeting cell surface components [21], [22]. Our group has been working for decades towards developing immunotherapeutic approaches to prevent or ameliorate disseminated, healthcare-associated fungal infections. These efforts have resulted in the initiation of a Phase I clinical trial of our anti-*Candida* vaccine that targets the Als3p, a known adhesin/invasion [23].

In our efforts to develop additional protective antigens against *Candida*, we have identified properties of the recombinant N-terminus of Hyr1p (rHyr1p-N) [12] that make it highly desirable for further development as both active and passive immunotherapy target. In our previous study, we demonstrated by using indirect immunofluorescence that Hyr1p is expressed on the cell surface of *C. albicans* hyphae [12]. These findings were further confirmed by our comparative indirect immunofluorescence of *C. albicans* using purified IgG raised against 8 hydrophilic peptides of rHyr1p-N pre- and post-absorption with *C. albicans* hyphae and control IgG (Figure 5A). We hypothesized that immunotherapies targeting the cell wall Hyr1p would have the dual benefit of the immune system recognizing the fungus and enhancing phagocyte killing of *Candida*. In this study, our data on rHyr1p-N has shown efficacy in animal models at doses 10–30 times less than those used for rAls3p-N (i.e. ∼50% survival for 10–33 µg dose for rHyr1p- N vs. 300 µg dose of rAls3p-N) [12], [19], [24]. Additionally, the mechanism of action appears to be considerably different from that of rAls3p-N. Rabbit polyclonal IgG raised against 8 different 14-mer peptides from regions of rHyr1p-N substantially protects mice from experimental disseminated candidiasis, whereas, our previous studies indicated that the mechanism of action of the rAls3p-N vaccine is dominantly dependent on T cells and anti-rAls3p-N antibodies are not the central mechanism of protection [19], [24]. Furthermore, the rHyr1p-N maintained its efficacy in the neutropenic mouse model. These findings suggest that Hyr1p is a promising target for both active and passive immunization.

Tissue fungal burden and histopathological examination of kidneys harvested from mice vaccinated with rHyr1p-N or alum alone further confirmed the efficacy of the rHyr1p-N vaccine. However, it appears that the histopathology difference between the control (Figure 2A) and rHyr1p-N vaccinated mice (Figure 2B) was more prominent than that of tissue fungal burden of the same organs. In this regard, it has been previously reported that colony counting can underestimate the tissue fungal burden in the presence of hyphae and pseudohyphae [25], [26], likely because tissue homogenization kills fungal filaments. We found that control mice had significantly more filamentous fungi in kidneys than vaccinated mice which had less abscesses mainly consisting of yeast form fungal elements. Therefore, tissue homogenization likely artificially lowers the colony counts for kidneys harvested from control mice but not from rHyr1p-N-vaccinated mice, making the difference less prominent.

Our results also show a dose response of anti-Hyr1p IgG in protecting mice from disseminated candidiasis. We confirmed that the protection elicited by anti-Hyr1p IgG was specific to Hyr1p since absorbed IgG with *C. albicans* hyphae lost its ability to protect mice against hematogenously disseminated candidiasis (Figure 5B). These results suggest that the mechanism of protection rendered by rHyr1p-N appears to be attributed, at least in part, to protective antibody response. Further studies to elucidate the role of T-cells vs. B-cells in the mechanism of rHyr1p-N protection against disseminated candidiasis are currently under active investigation.

In this study, we show that pooled IgG raised against 8 Hyr1 peptides directly neutralized the function of Hyr1p in resisting phagocyte killing rather than enhanced opsonophagocytosis. This is evident by the ability of F(ab′)_2_ fragments (prepared from anti-rHyr1p-N antibodies) to restore phagocyte killing of *C. albicans* overexpressing Hyr1p to levels equivalent to that of the suppressing strain (Figure 4D). However, the rHyr1p-N vaccine maintained its efficacy in neutropenic mice. This can be explained by the fact that cyclophosphamide induces leukopenia in mice with minimal effect on tissue phagocytes. Further experimentation is necessary to determine specific peptide(s) by which antibodies are generated to protect the host against disseminated candidiasis.

In summary, the rHyr1p-N vaccine is a promising candidate for further development. The vaccine is efficacious in both immunocompetent and immunocompromised mice, when mixed with alum as an adjuvant, against multiple clinical isolated strains of *C. albicans* [12], and against several non-*albicans Candida* species.

## Materials and Methods

### Candida strains and growth conditions

*C. albicans* 15663, *C. glabrata* 31028, *C. parapsilosis* 22019 and *C. tropicalis* 4243 are clinical bloodstream isolates collected from Harbor-UCLA Medical Center. *C. krusei* 91-1159 was generously provided by Michael Rinaldi, San Antonio, TX). *C. albicans* strains CAAH-31 and THE31 were engineered as described in our previous study and doxycycline was used to regulate the *HYR1* expression [12]. All tested strains were routinely grown in YPD (2% Bacto Peptone, 1% yeast extract, 2% dextrose). Cell densities were determined by counting in a hemacytometer.

### rHyr1p-N production

6×His tagged rHyr1p-N was produced in *E. coli* and purified by Ni-agarose affinity column as previously described [12]. Endotoxin was removed from rHyr1p-N using ProteoSpin Endotoxin Removal kit (Norgen Bioteck Corporation, Ontario, Canada), and the endotoxin level was determined with Limulus Amebocyte Lysate endochrome (Charles River Laboratories, Wilmington, MA) per manufacturer's instruction. Using this procedure, endotoxin was reduced to <0.1 EU per dose of the vaccine.

### Synthetic *peptides* and rabbit anti-Hyr1p polyclonal antibodies

Eight peptides derived from rHyr1p-N (Table 1) were commercially synthesized and used to generate anti-Hyr1p antibodies. Peptides were >85% pure as determined by HPLC and mass spectrometry (GenScript, Piscataway, NJ). They were conjugated to keyhole limpet hemocyanin (KLH) through additional cysteine from either N- or C- terminus before raising rabbit antiserum individually using a standard immunization protocol (GenScript, Piscataway, NJ). Total IgG from pooled serum was affinity purified using Pierce Protein A plus Agarose (Thermo Scientific, Rockford, IL) per the manufacturer's instruction prior to administering in passive immunization studies.

### Immunofluorescence detection of Hyr1p cellular localization

Indirect immunofluorescence was performed using pooled rabbit anti-Hyr1p IgG raised against 8 peptides of rHyr1p-N as previously described [12]. In brief, *C. albicans* blastospores (1×10^7^) were pre-germinated in RPMI 1640 for 90 min at 37°C and transferred into a 4-well chamber slide (Nalge Nunc International). After incubation at 4°C for 30 min, the cells were blocked with 300 µl of 1.5% mouse serum, then stained with 1∶500 dilution of either 1) pooled anti-Hyr1p IgG, 2) pooled anti-Hyr1p IgG absorbed with *C. albicans* hyphae (by incubating the pooled IgG repeatedly for 4 times with 1×10^7^ *C. albicans* hyphae for 30 min each time on ice), or 3) rabbit control IgG. The cells were counterstained with fluorescein isothiocyanate (FITC)-labeled goat anti-rabbit IgG at 1∶100 dilution prior to imaging with Zeiss *Axioskop* fluorescence microscopy.

### Immunization protocol and animal studies

All active vaccinations were conduced as previously described [12]. In brief, juvenile (10–12 week) Balb/C mice were vaccinated subcutaneously with 30 µg of rHyr1p-N mixed with alum (2% Alhydrogel; Brenntag Biosector, Frederikssund, Denmark) as an adjuvant in phosphate buffered saline (PBS) on day 0, boosted with the same dose on day 21, then infected via the tail vein on day 35 [27]. Control mice were vaccinated with alum alone.

To test the efficacy of the vaccine in immunocompromised mice, mice were vaccinated as above prior to inducing neutropenia by intraperitoneal injection of 200 mg/kg of cyclophosphamide on day −2 followed by another dose of 100 mg/kg on day +7 relative to infection. This regimen results in approximately 10 days of leucopenia with reduction in neutrophil, lymphocyte and monocyte counts, as described previously [28], [29], [30]. For both immunocompetent and neutropenic mice differences in survival between vaccinated and adjuvant vaccinated mice were compared by the Log Rank test.

For passive immunization, immune IgG was administered intraperitoneally to naïve mice 2 hr before infecting intravenously with *C. albicans*. Control mice were given non-specific, rabbit IgG (Innovative Research, USA). IgG doses were repeated 3 days after infection, and survival of mice was monitored twice daily.

Quantitative culturing of kidneys from vaccinated or control mice infected with different species of *Candida* was performed as previously described [31]. In brief, mice were infected through tail veins. Kidneys were harvested 3 days post infection, homogenized, serially diluted in 0.85% saline, and quantitatively cultured on YPD that contained 50 µg/ml chloramphenicol. Colonies were counted after incubation of the plates at 37°C for 24 to 48 hr, and results were expressed as log CFU per gram of infected organ.

Concomitant with the fungal burden experiment, kidneys were removed aseptically from two mice per group for histopathological examination. Kidneys were immersed in zinc formalin fixative until examination. Fixed organs were dehydrated in graded alcohol solutions, embedded in paraffin, and cut into 6-µm-thick sections. Mounted sections were stained with Gomori methenamine silver and examined by light microscopy [32].

### Enzyme-linked immunosorbent assay (ELISA)

To test if the rHyr1p-N vaccine induced an immune response, antibody titers of serum samples collected from vaccinated and control mice were determined by ELISA in 96-well plates as previously described [27]. Wells were coated at 100 µl per well with rHyr1p-N at 5 µg/ml in PBS. Mouse sera were incubated for 1 hr at room temperature following a blocking step with Tris-buffered saline (TBS; 0.01 M Tris HCl [pH 7.4], 0.15 M NaCl) containing 3% bovine serum albumin. The wells were washed three times with TBS containing 0.05% Tween 20, followed by another three washes with TBS. Goat anti-mouse secondary antibody conjugated with horseradish peroxidase (Sigma) was added at a final dilution of 1∶5000, and the plate was further incubated for 1 hr at room temperature. Wells were washed with TBS and incubated with substrate containing 0.1 M citrate buffer (pH 5.0), 50 mg of *o*-phenylenediamine (Sigma), and 10 µl of 30% H_2_O_2_. The color was allowed to develop for 30 min, after which the reaction was terminated by addition of 10% H_2_SO_4_ and the optical density (OD) at 490 nm was determined in a microtiter plate reader. Negative control wells received only diluent, and background absorbance was subtracted from the test wells to obtain final OD readings. The ELISA titer was taken as the reciprocal of the last serum dilution that gave a positive OD reading (i.e., more than the mean OD of negative control samples plus 2 standard deviations).

### F(ab′) _2_ blocking assay

To study the mechanism of protection mediated by anti-Hyr1p antibodies in phagocyte-mediated killing of *C. albicans*, HL-60 cells that have been differentiated to neutrophil-like phenotype were used [12]. Killing assay was conducted in the presence of anti-Hyr1p IgG or F(ab′) _2_ fragments as described before [12]. In brief, HL-60 cells were induced with 2.5 µM of retinoic acid and 1.3% DMSO for 3 days at 37°C with 5% CO_2_. Immune anti-Hyr1 peptides (Table 1) sera were pooled and total IgG was isolated using protein A agarose (Thermo Scientific). Serum collected from the same rabbits prior to immunization with the peptides served as control serum. The F(ab′) _2_ fragments from immune or control IgG were purified with Pierce F(ab′)_2_ Preparation Kit according to the manufacturer's instruction. SDS-PAGE analysis indicated >95% of Fc fragment was digested by this kit (data not shown). Next, *C. albicans* cells overexpressing or suppressing Hyr1p [12] were incubated with 50 µg/ml of vaccinated or control F(ab′) _2_ fragments on ice for 45 min. *C. albicans* cocultured with the F(ab′) _2_ fragments were incubated with HL-60 derived neutrophils for 1 hr at 37°C with 5% CO_2_ prior to sonication and quantitative culturing on YPD plates. % killing was calculated by dividing the number of CFU after coculturing with HL-60 derived neutrophils by the number of CFU from *C. albicans* incubated with media without HL-60 derived neutrophils.

### Statistical analysis

The nonparametric log rank test was used to determine differences in the survival times of the mice. Neutrophil killing assay, titers of antibody, and tissue fungal burden were compared by the Mann-Whitney U test or Wilcoxon rank sum test for unpaired comparisons. Correlations were calculated with the Spearman rank sum test. *P* values of <0.05 were considered significant.

All procedures involving mice were approved by the Los Angeles Biomedical Research Institute animal use and care committee for the project 11672-05 specifically to this vaccine study, following the National Institutes of Health guidelines for animal housing and care. The institute has a US Public Health Service approved animal welfare assurance number A3330-01.
